# RGO/WO_3_ hierarchical architectures for improved H_2_S sensing and highly efficient solar-driving photo-degradation of RhB dye

**DOI:** 10.1038/s41598-021-84416-1

**Published:** 2021-03-03

**Authors:** Swati S. Mehta, Digambar Y. Nadargi, Mohaseen S. Tamboli, Thamraa Alshahrani, Vasudeva Reddy Minnam Reddy, Eui Seon Kim, Imtiaz S. Mulla, Chinho Park, Sharad S. Suryavanshi

**Affiliations:** 1 School of Physical Sciences, PAH Solapur University, Solapur, MS 413255 India; 2 School of Chemical Engineering, Yeungnam University, 280 Daehak-ro, Gyeongsan, 38541 Republic of Korea; 3 Department of Physics, College of Science, Princess Nourah Bint Abdulrahman University, Riyadh, 11671 Saudi Arabia; 4 Former Emeritus Scientist (CSIR), Centre for Materials for Electronics Technology, Pune, 411008 India

**Keywords:** Energy science and technology, Materials science, Nanoscience and technology

## Abstract

Surface area and surface active sites are two important key parameters in enhancing the gas sensing as well as photocatalytic properties of the parent material. With this motivation, herein, we report a facile synthesis of Reduced Graphene Oxide/Tungsten Oxide RGO/WO_3_ hierarchical nanostructures via simple hydrothermal route, and their validation in accomplishment of improved H_2_S sensing and highly efficient solar driven photo-degradation of RhB Dye. The self-made RGO using modified Hummer’s method, is utilized to develop the RGO/WO_3_ nanocomposites with 0.15, 0.3 and 0.5 wt% of RGO in WO_3_ matrix. As-developed nanocomposites were analyzed using various physicochemical techniques such as XRD, FE-SEM, TEM/HRTEM, and EDAX. The creation of hierarchic marigold frameworks culminated in a well affiliated mesoporous system, offering efficient gas delivery networks, leading to a significant increase in sensing response to H_2_S. The optimized sensor (RGO/WO_3_ with 0.3 wt% loading) exhibited selective response towards H_2_S, which is ~ 13 times higher (R_a_/R_g_ = 22.9) than pristine WO_3_ (R_a_/R_g_ = 1.78) sensor. Looking at bi-directional application, graphene platform boosted the photocatalytic activity (94% degradation of Rhodamine B dye in 210 min) under natural sunlight. The RGO’s role in increasing the active surface and surface area is clarified by the H_2_S gas response analysis and solar-driven photo-degradation of RhB dye solution. The outcome of this study provides the new insights to RGO/WO_3_ based nanocomposites’ research spreadsheet, in view of multidisciplinary applications.

## Introduction

To conquer over global challenges like water and air pollution rose due to the escalating economic development, there is an immense demand for the nanomaterials with multifunctional properties. Tungsten oxide (WO_3_) and its composites are considered to be potential candidates for multifunctional applications^1^. WO_3_ is n-type wide band gap (2.4–2.8 eV) metal oxide semiconductor having fascinating properties like high chemical and physical stability, non-toxicity, abundant sources, and low cost. Ultimately, it has a wide spectrum of applications such as electrochromic devices, photocatalysis, batteries, supercapacitors and gas sensors^2–6^.

Speaking about the prime motivation of conducting the present work, pristine WO_3_ is not competitive enough for multifunctional application, as is. For example, the lower level of WO_3_ conduction band does not have enough capacity to react with strong electron acceptors, when applying for photocatalytic application. This leads directly to a fast recombination, and thereby a low photocatalytic performance^7^. On the other side, there is a plenty of room to enhance/improve the gas response characteristics of pristine WO_3_ in particular, and any metal oxide in general, via -(i) doping/incorporating the conducting channels, (ii) spillover effect, (iii) forming hetero/homo-junction, and iv) increasing the surface area for more gas diffusion^8–11^.

The material like Reduced Graphene Oxide (RGO) will serve the two purpose (enhancing the gas response as well as photocatalytic performance) in one go, due to its excellent electrical properties, exceptional thermal conductivity (5000 W/mK), robust mechanical strength, large surface area, and scalable production. Its conductive channels are the strong receiver and reservoir of the photo-generated electrons. It could greatly facilitate the isolation of photo-induced charges, stimulate the transition of interface charges, and increase the lifespan of pairs of electron–hole photogenerates. The photocatalysis of graphene/WO_3_ nanocomposites, therefore, increases remarkably. While in gas response, RGO -(i) increases surface area by improving S_BET_, (ii) provides additional active sites for adsorption of test gas, and (iii) increases charge transport which results in better response for different test gases^12^.

The current research work exemplifies the systematic efforts in tuning the aforementioned properties of pristine WO_3_ using RGO incorporation in such a way that it can be used bi-directionally as an excellent gas sensor and efficient photocatalyst for dye degradation under natural sunlight.

## Experimental

**Scheme 1:**
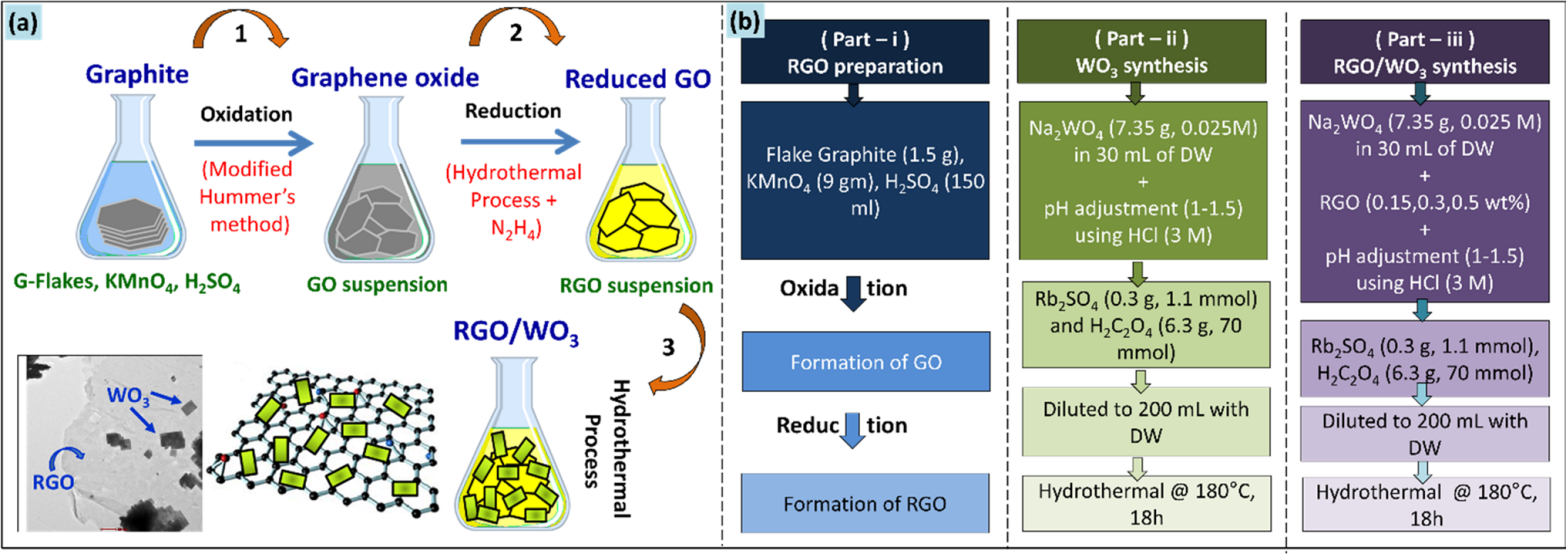
(**a**) Complete synthesis steps of RGO/WO_3_ nanocomposites, (**b**) Set of samples’ flow chart.

The chemicals used in a representative synthesis were: graphite flakes, potassium permanganate (KMnO_4_), sulfuric acid (H_2_SO_4_), sodium tungstate (Na_2_WO_4_), hydrochloric acid (HCl), oxalic acid (H_2_C_2_O_4_), and rubidium sulfate (Rb_2_SO_4_). All the reactants were AR-grade class, obtained from Sigma Aldrich, and used as received. For the entire synthesis process, double distilled water (DW) was used. Scheme 1 provides a condensed schematic that describes the whole synthetic protocol (Scheme 1a), along with the planning of the relevant sample classes described in this article (Scheme 1b).

### RGO preparation

RGO was synthesized in the first part using the modified Hummer method^13^, where the graphite flakes were chemically oxidized. As the oxidation of graphite is exothermic process, the reaction individuals (Graphite flakes, KMnO_4_, H_2_SO_4_) were cooled down (~ 10–15 °C) prior to actual reaction. To be at better side (and needlessly), the Teflon liner which the reaction was planned, was also cool down to the same temperature of the reactants. The refrigerated graphite (1.5 g) and KMnO_4_ (9 gm) were transferred into the Teflon lined vessel, followed by the addition of H_2_SO_4_ (150 ml). As soon as the H_2_SO_4_ was added, the oxidation of graphite started. The Teflon liner was covered with its airtight lid and inserted in the stainless steel autoclave. The autoclave was then fixed firmly and placed in the refrigerator at 0–4 °C for 1.5 h. This step of cooling the reaction mixer is favored to obtain the uniform and minimal/thin layers of GO nanosheets. Finally, the reduction process for obtaining the RGO, autoclave was heated to 100 °C for 1.5 h. The obtained black slurry was treated with hydrazine hydrate solution (30% N_2_H_4_) upon constant stirring, till the color of the slurry turns into golden yellow. The mixture was then washed with HCl (2 M), followed by double distilled water, till the suspension pH becomes 7. The suspension was eventually filtered and dried at 80 °C to generate dark brown/black reduced graphene oxide.

### RGO/WO_3_ synthesis

In the second part, desired amount of GO was dispersed in 10 mL ethanol/water mixture by ultrasonicating the suspension for 2 min, only. The extended ultrasonication is not favored to prevent the nanosheets from tearing off. As obtained GO suspension was added to separately prepared aqueous solution of sodium tungstate (7.35 gm, 0.025 M) under constant stirring. The solution was acidified to pH 1–1.5 using HCl (3 M), to obtain the lemon yellow colored precipitate. Complexing surfactants Rb_2_SO_4_ (0.3 g, 1.1 mmol) and H_2_C_2_O_4_ (6.3 g, 70 mmol) were then added to the mixture and diluted to 200 mL using distilled water. A translucent, homogeneous and stable GO/WO_3_ sol was obtained. This solution was transferred to 250 mL Teflon contained autoclave, sealed, and maintained at 180 °C for 18 h. Autoclave was allowed to cool down naturally, and the product was filtered, washed, and dried at room temperature (27 °C) followed by annealing at 400 °C in air for 2 h. For the control experiment (pristine WO_3_), the aforementioned procedure was followed without an addition of GO. The samples were labelled as “G0”- for pristine WO_3_, and “G1, G2, G3”- for 0.15, 0.3 and 0.5 wt% of GO loaded WO_3_.

### Measurements

The crystal structures of as-synthesized materials were characterized by X-rays diffractions (XRD) with the incident of radiation (λ = 1.5406 Å, scan rate = 0.01°/s, scan range = 10–80°) at D_max_ 2550 V X-ray (Rigaku, Tokyo, Japan) diffractometer. The field emission scanning electron microscopy (FESEM, Bruker XFlash 6130) and transmission electron microscopy (TEM, FEI Techni, G2, 300 kV) were used to investigate the microstructural and grain size evolution of the developed hierarchical assembly. In order to revalidate the crystal structure obtained from XRD, high-resolution electron microscope (HRTEM) and selective area electron diffraction (SAED) images were used. The specific surface area was determined by the Quantachrome Instruments ST-2000 surface and pore size analyser. The distribution of the pore size was calculated using the BJH method from the adsorption/desorption isotherms.

#### Sensor fabrication and test method

The study of gas response was performed using an indigenous 1 L capacity gas sensor chamber (presented in our earlier reports), built commercially^14^. The thick sensor films were produced with screen printing technique. For this purpose, the active material paste was formulated into the temporary binding agents (ethyl cellulose and butyl carbitol acetate). In the paste formulation, the proportion from inorganic to organic was held at 70:30 vol%. The paste was then moved to the mesh for screen printing and squeegeed on the pre-cleaned alumina substrates. These thick films were dried at room temperature, followed by sintering at 400 °C for 1 h, to eliminate the binder. Electrode pattern was prepared by using silver paste for the electrical measurements.

The sensor temperature can be controlled up to 400 °C with ± 2 °C precision. The as-prepared sensor thick film was fixed tightly to the two-probe sample holder. This assembly goes into the tubular furnace with the coaxially fitted glass chamber. At different operating temperatures, the sensor thick films were analyzed for various test gases. The sensor response (S) was specified in Ra/Rg, where Ra and Rg are the sensor resistance values, which are determined in both the air and the gas atmosphere of the test. The various test gases like acetone, ammonia, ethanol, trimethylamine, xylene, propanol, and diethanolamine and hydrogen sulphide gas, were analysed.

#### Photocatalytic activity test

Rhodamine-B dye degradation was evaluated for photocatalytic activity of RGO/WO_3_ nanostructures. A photocatalyst powder (0.3 g) was added to Rhodamine B aqueous solution (5 mg/L, 100 mL) at room temperature to prepare the reaction suspension. The suspension was constantly mixed for 60 min in the dark before putting it under natural sunlight. The solution was finally placed in a magnetically stirred beaker and irradiated under the natural sunlight. On a digital lux meter, the average intensity of natural sunlight was measured and 8.95 × 104 lx was found. Irradiated sample aliquots at a specific time interval were reserved, Rhodamine B degradation was determined using UV–Vis analysis (Cary UV-60 Spectrophotometer, Agilent Technologies). Following a degradation of the dye, the maximum absorption band of Rh B at 553 nm gets changed. The degradation efficiencies of the dyes were estimated by the equation:

1 $$\eta (\% ) = \frac{{(C_{i} - C_{f} )}}{{C_{i} }} \times 100$$

where C_i_ and C_f_ are the dye concentration in solution before and after irradiation. Furthermore, the other parameters like Rhodamine B concentration, the irradiation time effect and the degradation efficiency, were studied.

## Results and discussion

**Figure 1:**
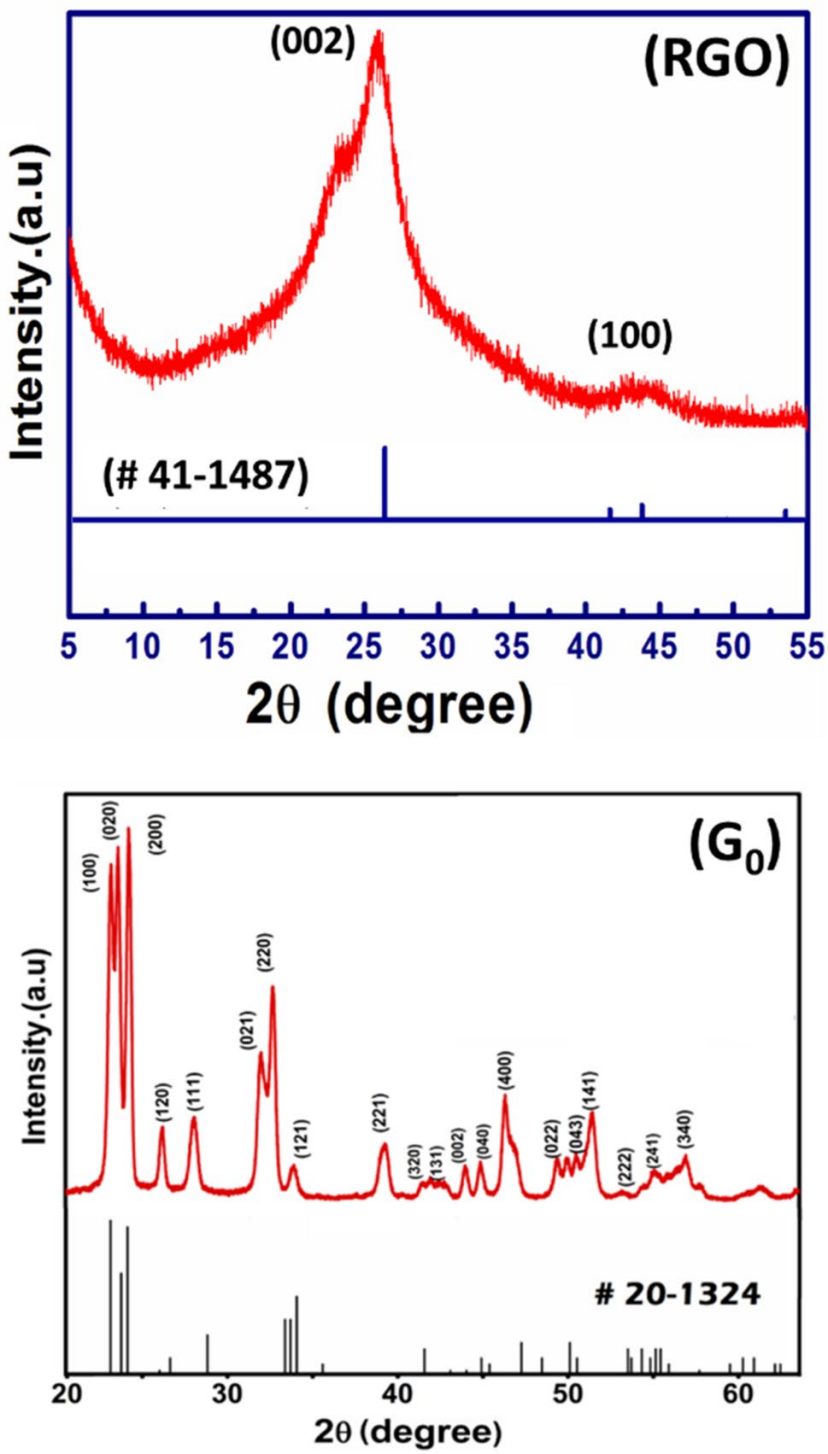
XRD signatures of self-developed (RGO), and pristine WO_3_ (G0).

The crystallinity and phase formation of the developed material was characterized by XRD technique as shown in Fig. 1.

**Figure 2:**
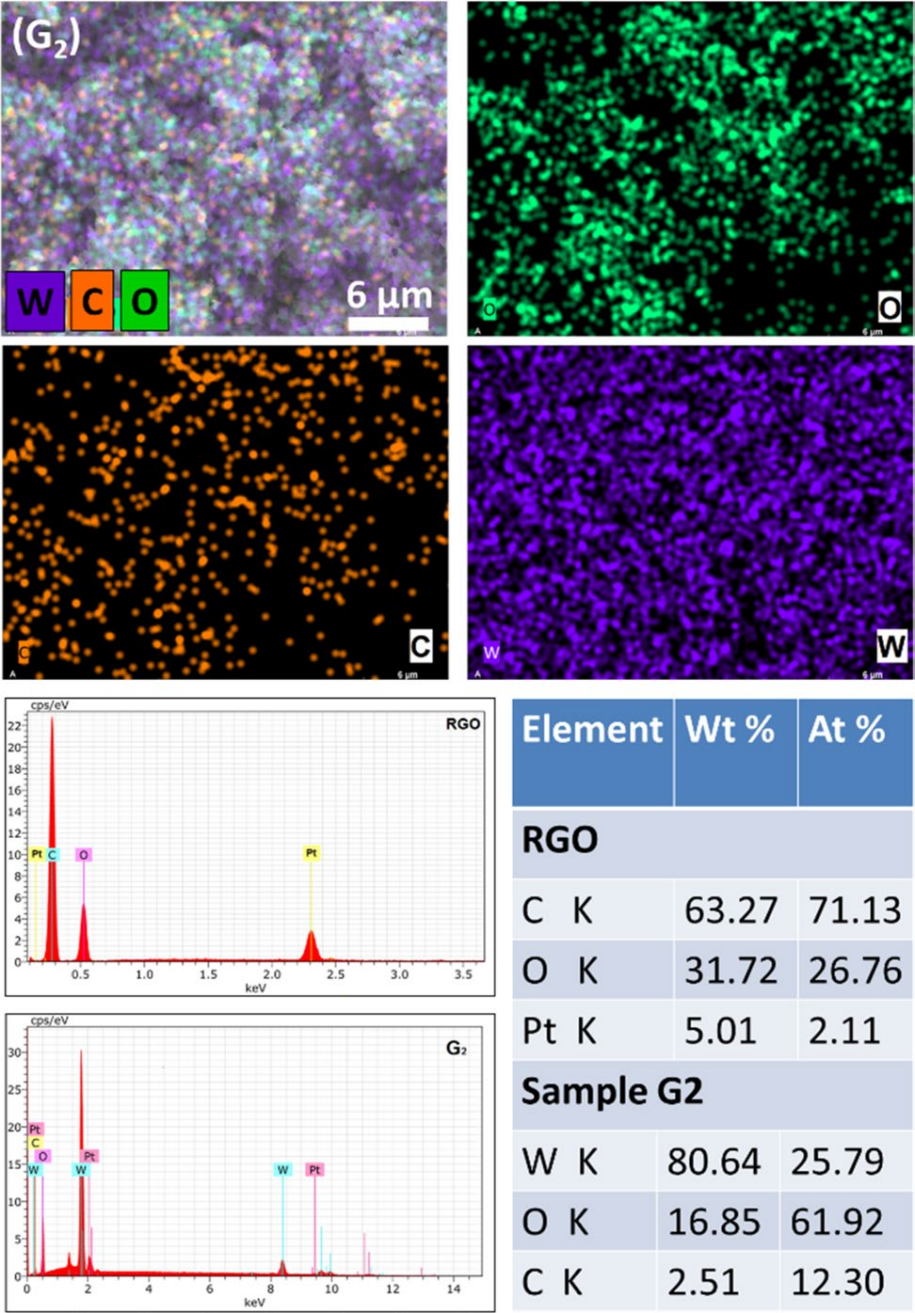
Elemental mapping of sample G2, and EDX spectra of RGO and G2 sample with analysis table.

In the XRD signature of as-developed RGO, the concentrated peak at ~ 26° of 2θ clearly demonstrates the self-made RGO is in well agreement to those obtained from commercial sources. The primary diffraction peak at 25.9° and secondary peak at 43.8° corresponds to (002) and (100) plane of graphite, respectively (JCPDS-Card No. 41-1487). This shows that the desired RGO preparation by graphite oxidation is very promising. The XRD spectrum of the developed WO_3_ (sample G0) authorizes the orthorhombic crystal structure. Though all RGO/WO_3_ samples (G1–G3) show the same crystal structure (orthorhombic), their XRD comparison is highlighted in the supplementary section for the more clarity (see SI-I). However, the crystallite size and lattice parameters are tabulated over here in Table 1. The smaller crystallite sizes (10.8–10.9 nm), are undoubtly giving the impression of a possible applicant for improved gas sensing and photocatalytical property, since the smaller crystallite size provides a greater surface area, which increases the likelihood of adsorption/desorption or interaction^15^. From the Table 1 (and as per the expectation), the developed RGO/WO_3_ nanocomposites show no influence of RGO loading in WO_3_ matrix. The XRD comparison graphs (see SI-I) also revalidates the no profound influence of RGO (0.15 to 0.5 wt%) on the crystal structure and plane orientations. In addition, comparatively less loading amount as well as low diffraction intensity of graphene, shows no distinguished peaks with respect to WO_3_ characteristic peaks^16^. The homogeneous distribution and presence of RGO in WO_3_ structure (sample G2) are illustrated in the elemental mapping and EDS spectrum (Fig. 2). Further, the EDX spectra of RGO confirm presence of carbon element with prominent high intensity peak of carbon. The EDX spectra of sample G2 illustrates the strong signals of elementals W, O with C indicating the phase purity of synthesized material. Elemental distribution in the EDS spectrum designates the compositional ratio of W:O is almost 1:3, which is in line with the stoichiometric percentage of WO_3_. The elemental mapping of sample G2 showcase the uniformity in the distribution of graphene. The EDAX analysis of sample G0-G4 indicates the presence of W, O and C are in the atomic proportions with the initial precursor amounts taken (See SI-II, Table S1). In Fig. 2, the atomic ratios of the elements of RGO and sample G2 are shown in percentage (see inset table). The EDS graph (left side) showcase the respective elements of the sample in accordance with their amounts. For more clarity, the EDS spectrum of samples G0, G1, and G3 are highlighted in the supplementary section (SI-III).

**Table 1:** Sample id, crystallite size and the lattice parameters of the developed RGO/WO_3_.

Sample id	Crystallite size (nm)	Lattice parameters (Å)
G0	10.8	a = 3.86, b = 7.54, c = 7.32
G1	10.8	a = 3.86, b = 7.54, c = 7.32
G2	10.9	a = 3.86, b = 7.54, c = 7.32
G3	10.9	a = 3.86, b = 7.54, c = 7.32

**Figure 3:**
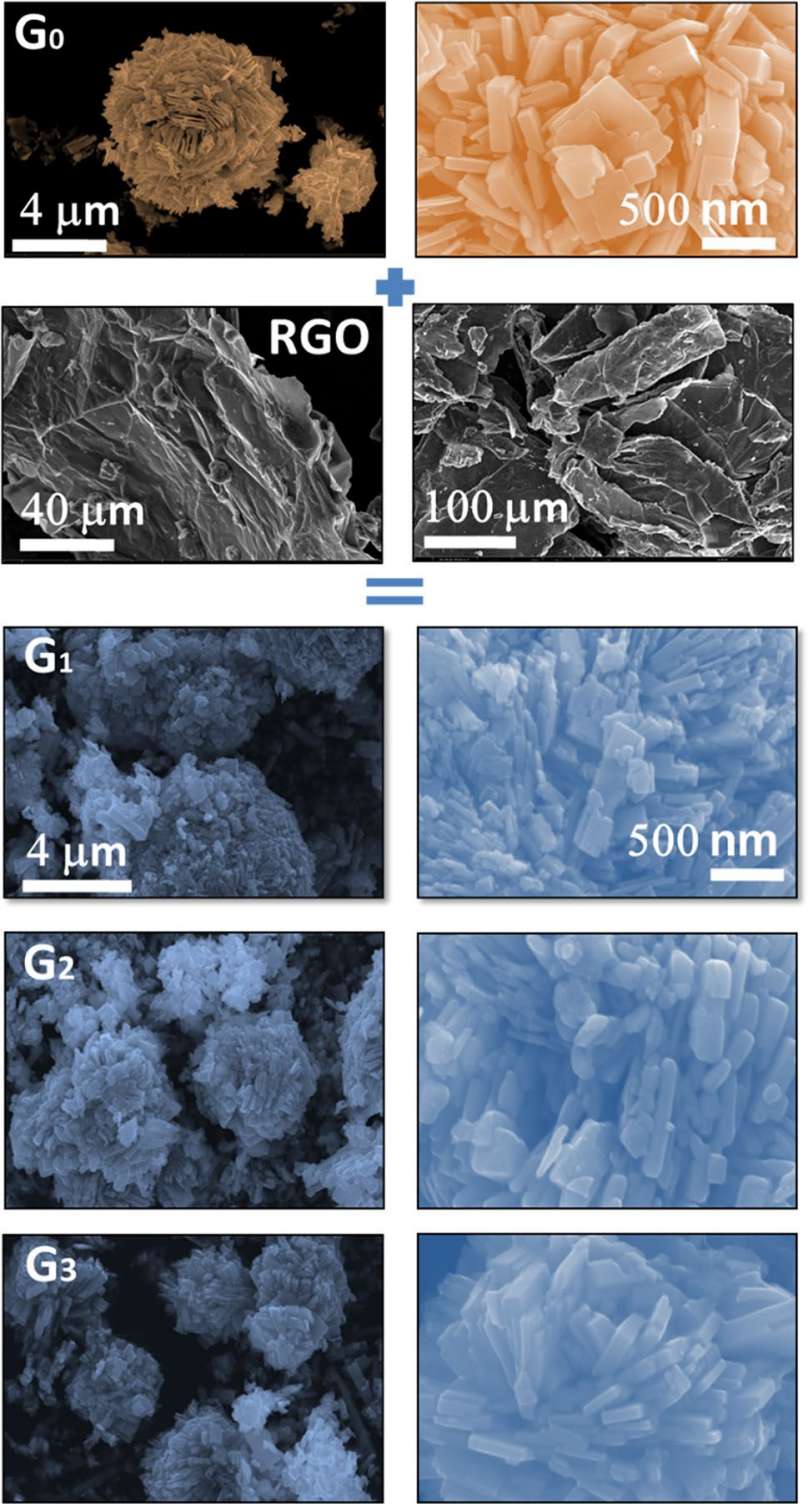
FESEM images of pristine (G0) and RGO/WO_3_ (G1–G3), samples along with self-made RGO.

**Figure 4:**
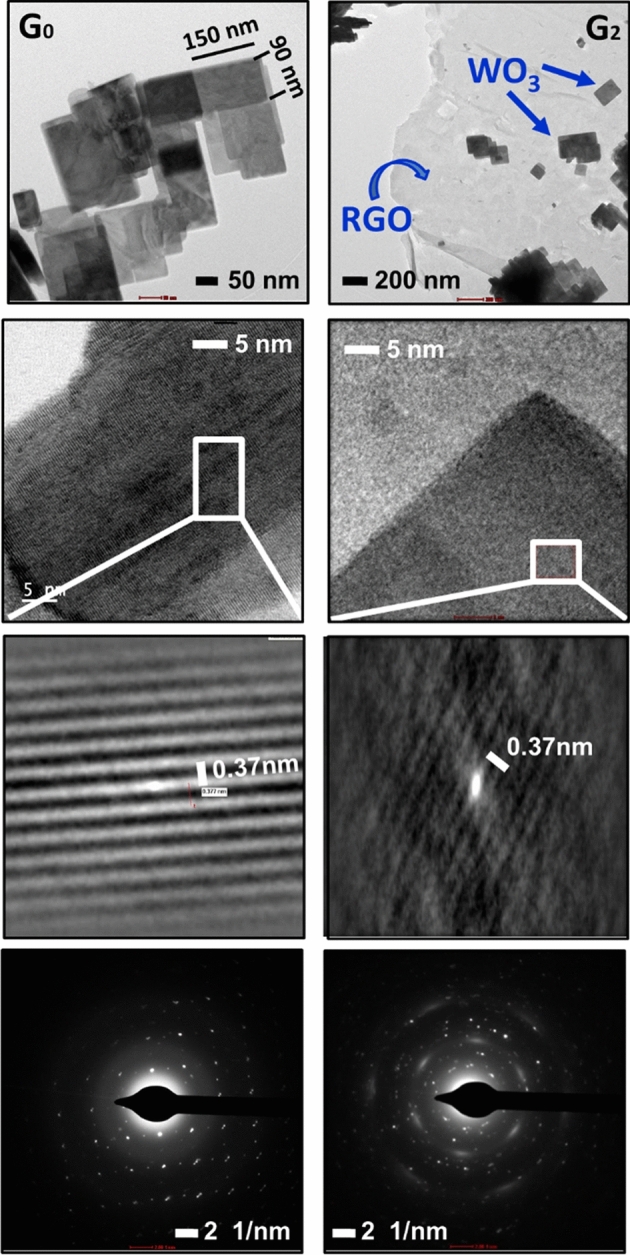
TEM images of low and high magnification with HRTEM and SAED pattern of pristine and 0.3% RGO/WO_3_ samples.

The hierarchical architectures of RGO/WO_3_, developed by facile hydrothermal technique, are highlighted in Figs. 3 and 4. The FESEM images of pristine and RGO/WO_3_ materials (Fig. 3) shows marigold microstructure, having composed of numerous nanoplates (see magnified FESEM images) got arranged in such a way that the stable morphology appears to be a marigold flower. These nanoplates are having the dimensions ~ 90–160 nm in length, ~ 85–95 nm in width, with the third dimension ~ 20–30 nm thick. The nanoplates are well resolved in the TEM analysis (Fig. 4). Speaking about RGOs morphology, its FESEM clearly shows the layered structure of carbon. The natural wrinkles in the carbon layers are seen due to their Van der Waals interactions, where the agglomeration is understandable at elevated temperature. Upon incorporation of these wrinkled layers (i.e. RGO) in the WO_3_ matrix, a slight change in the morphology is observed. Though, the parent microflower structure is preserved, the gradual change in the diameter of the microflower with disturbed compactness is seen. With increasing RGO content, the ability of forming bigger rounds is ceased, which is in the fitness of the natural behavior. Graphene sheets might act as separators between two WO_3_ moieties. The subsequent loosening of nanoplates is highlighted by different FE-SEM image which is shown in supplementary information (SI-IV). The unique and controlled morphologies observed in the present RGO/WO_3_ samples are likely to make promising materials as gas sensors and photocatalysts.

The microstructure of pristine WO_3_ (sample G0) and RGO/WO_3_ nanocomposite with 0.3 wt% of graphene (sample G2) are analyzed under TEM (Fig. 4). As discussed in the FE-SEM section, these nanoplates have the dimensions of length ~ 90–160 nm, and width ~ 85–95 nm. They are highly uniform, with high aspect ratio (Fig. 4). The nanoplates shape and uniformity remain undisturbed by the incorporation of RGO in the WO_3_ matrix. These nanoplates got well-dispersed over the thin graphene sheet, which is clearly visualized in the TEM image. These results are in well agreement with the elemental mapping studies under EDS analyses. HRTEM images show the lattice fringes spacing to be around 0.37 nm, equivalent to an o-WO_3_ plane (1 0 0). These nanoplates grown along a-direction of the lattice plane. The SAED pattern shows the transformation of single crystalline nature to polycrystalline, as RGO was incorporated in the WO_3_ matrix.

Let us now discuss the growth mechanism of RGO/WO_3_ nanocomposites. At the outset, the H_2_WO_4_ form on the RGO surface when an acid solution is added. H_2_WO_4_ is transformed into the WO_3_ crystal nucleus in the hydrothermal atmosphere, and promotes the nucleation of the WO_3_. The RGO sheets have baseline planes embellished by the groups of epoxy and hydroxyl, while groups of carbonyl and carboxy occur at borders^15^. All these groups act as anchor sites to support the building of RGO/WO_3_ nanoplates. Furthermore, hydrothermal reductions at high temperature and pressure can be seen as an effective way of re-establishing sp^2^ hybridized network^17^. At this supercritical temperature and pressure condition, the rupture of non-reactive oxygen comprising moieties occur^18^. As the amount of graphene increases, it implies a significant interaction between WO_3_ nuclei and graphene plate. This population density leads to the growth of crystal into nanoparticles. Additionally, graphene has brittle nature, and very delicate at elevated temperature. Thus, the formation of irregular nanoparticles is obvious. As a result, disturbed compactness of the RGO/WO_3_ microflower structure is observed in the present case, as the RGO was incorporated in the WO_3_.

**Figure 5:**
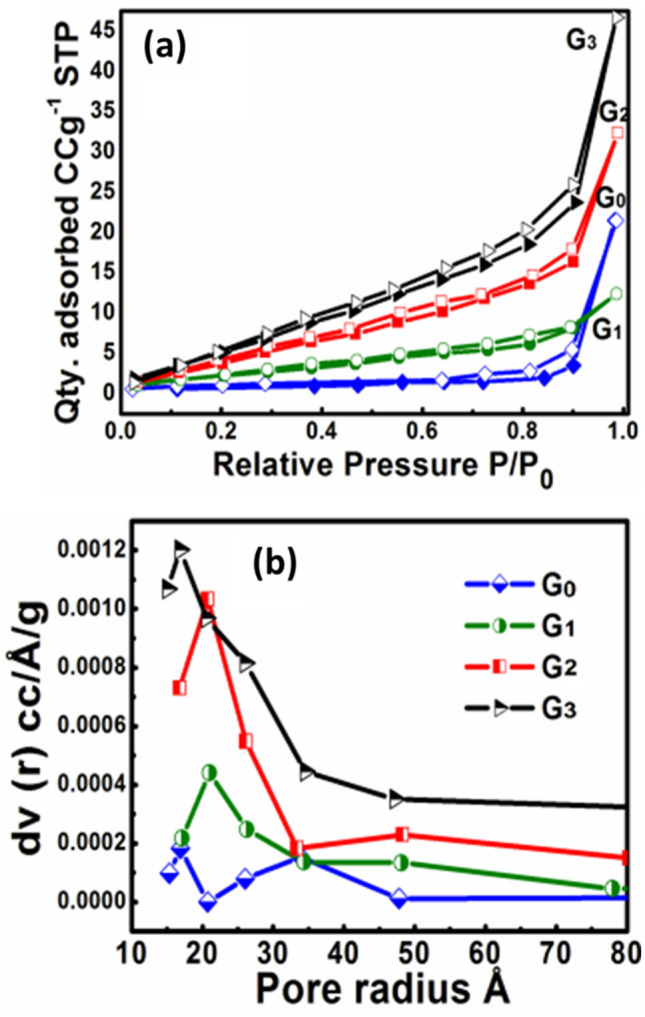
BET analysis curves (**a**) N_2_ absorption/desorption isotherms, and (**b**) Pore size distribution of samples G0–G3.

To obtain the surface area and pore size distribution of as-developed RGO/WO_3_ nanomaterials, nitrogen multilayer adsorption/desorption (BET) measurements were performed (Fig. 5). The process encapsulates the calculation of external as well as pore areas to assess the overall specific surface area, which provides crucial knowledge in investigating the effects of surface porosity and particle size, in the present case of dual applications (gas sensor and photocatalysis).

A hysteresis loop of isothermal adsorption–desorption of N_2_, ranges in the relative pressure (P/P_0_) of 0.1–0.9 for each sample. The obtained hysteresis loops are representative of type IV isotherm, with the form H_3_ and H_4_ loop-type. Type H_3_ loop represents loose groupings of plate-like particles, that form slit pores. This ultimately indicates that the sample G0 and G1 are composed of mesoporous microstructure. After increase in the amount of graphene the mesoporous structure is retained with H_4_ type of hysteresis loop, indicating a finite multilayer formation corresponding to complete filling of the capillaries and wide distribution of pore sizes. Table 2 shows Average pore radius (Å), Pore volume (cm^3^/g), and surface area (m^2^/g) of pristine and RGO incorporated WO_3_ samples.

**Table 2:** Average pore radius, Pore volume and Surface area of pristine and RGO incorporated WO_3_ samples.

Sample id	Avg. pore radius (Å)	Pore volume (cm^3^/g)	Surface area (m^2^/g)
G0	34.19	0.034	3.28
G1	20.90	0.018	6.61
G2	20.83	0.047	13.86
G3	20.77	0.069	20.04

### Gas sensing studies

**Figure 6:**
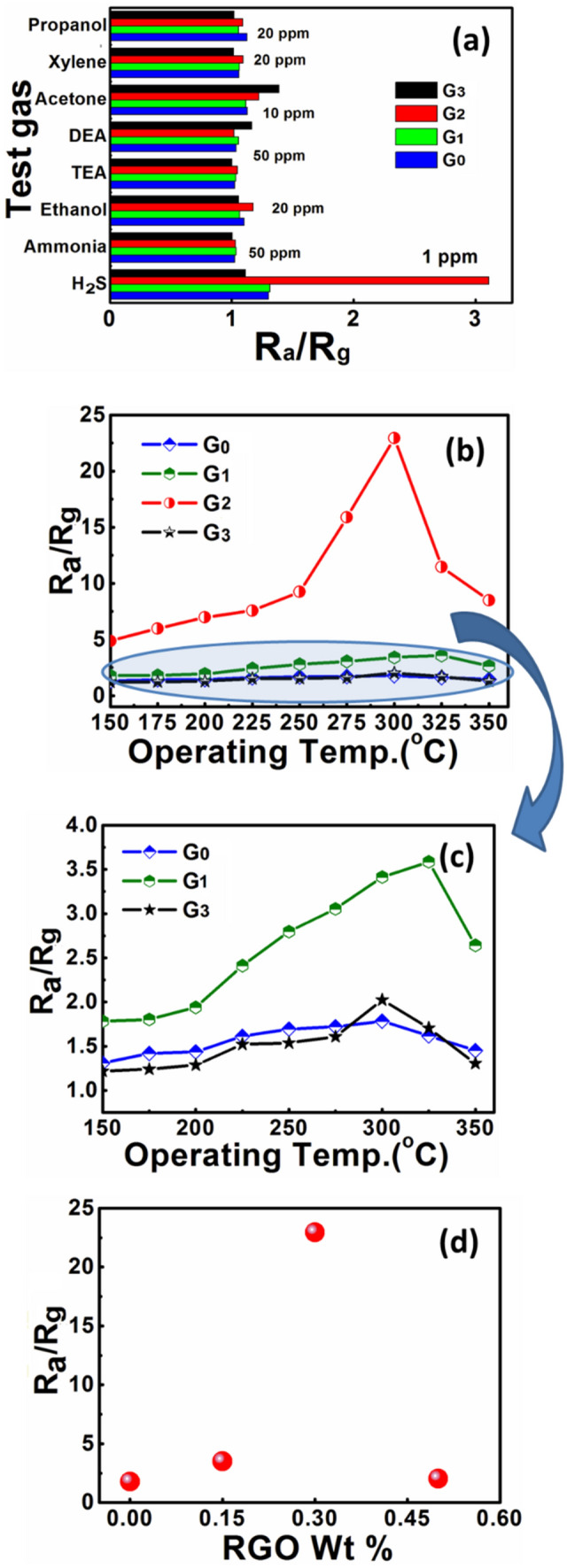
(**a**) Comparative analysis sensor response towards various test gases at 300 °C, (**b** and **c**) Sensor response as a function of operating temperature for pristine WO_3_ and RGO/WO_3_ samples towards 100 ppm H_2_S, respectively, (**d**) Effect of RGO loading on H_2_S sensing.

From the physico-chemical analyses, the developed RGO/WO_3_ nanocomposites with hierarchical morphology could be very promising material for gas sensing response due to their large effective surface area and great surface activity. This unique and hierarchical microstructure encourages us to further investigate gas sensing performance of developed RGO/WO_3_ nanomaterial. They found very efficient towards various reducing gases such as Propanol, Xylene, Acetone, Diethanolamine, Trimethylamine, Ethanol, Ammonia, and Hydrogen sulphide. This proficiency was explored systematically and illustrated in Fig. 6a. Amongst the response values (Ra/Rg) of all the test gases, sample G2 showed Ra/Rg = 3.11 response towards H_2_S at mere concentration of 1 ppm, at 300 °C operating temperature. Compared to other test gases, the obtained response is more than double, even at lower concentrations, at the same operating temperature. The investigations were, therefore, focused towards H_2_S gas sensing application.

In the optimum operating temperature studies of pristine and RGO incorporated WO_3_, all the sensors exhibited a distinct hump at an optimum operating temperature (see Fig. 6b). The hump is clearly visualized for sensor G2 in comparison to sensor G0, G1, and G3, due to large difference in the response magnitude. To explore it in more detailed manner, the graph for sensor G0, G1, and G3 is extrapolated in Fig. 6c, where the sensors shows -(i) the increased response, (ii) attain a peak value, and (iii) eventually drops with increasing operating temperature. The enhancement in the gas response of pristine WO_3_ after incorporating the graphene sheets, is due to the fact that -(i) increased conducting channels, and (ii) increased surface area.

In case of sensor G2, the fourfold increment in the surface area, led to optimal incorporation of RGO in the WO_3_ matrix. The loosely organized porous structure offers plentiful passages for effective and quick diffusion of H_2_S gas^1^. Furthermore, for the improved gas sensing competence, the WO_3_ board like morphology is useful in creating a bound film of RGO and WO_3_^5^. From the aforementioned studies, it is very clear that, though the higher dose of RGO incorporation overshoots the surface area, the proper amount of RGO incorporation plays a vital role in sensing H_2_S or any other test gas. Figure 6d demonstrates the H_2_S response values of the sensors at various RGO dosing; where remarkable improvement in the response is observed for 0.3 wt% of RGO in WO_3_. The Ra/Rg value shoot-up to 22.9 from 1.8, for 100 ppm H_2_S at 300^○^C of operating temperature. A significant influence on the performances of sensors is observed after uniform addition of WO_3_ to RGO planes. The WO_3_/RGO active surfaces have also the profound influence on performance of the chemical sensors. The response got decreased to 2.02 after further increase in the RGO loading to 0.5 wt%. It is due to the fact that large amount of RGO increases number of holes, which indicates the p-type behavior of the sensing element.

**Figure 7:**
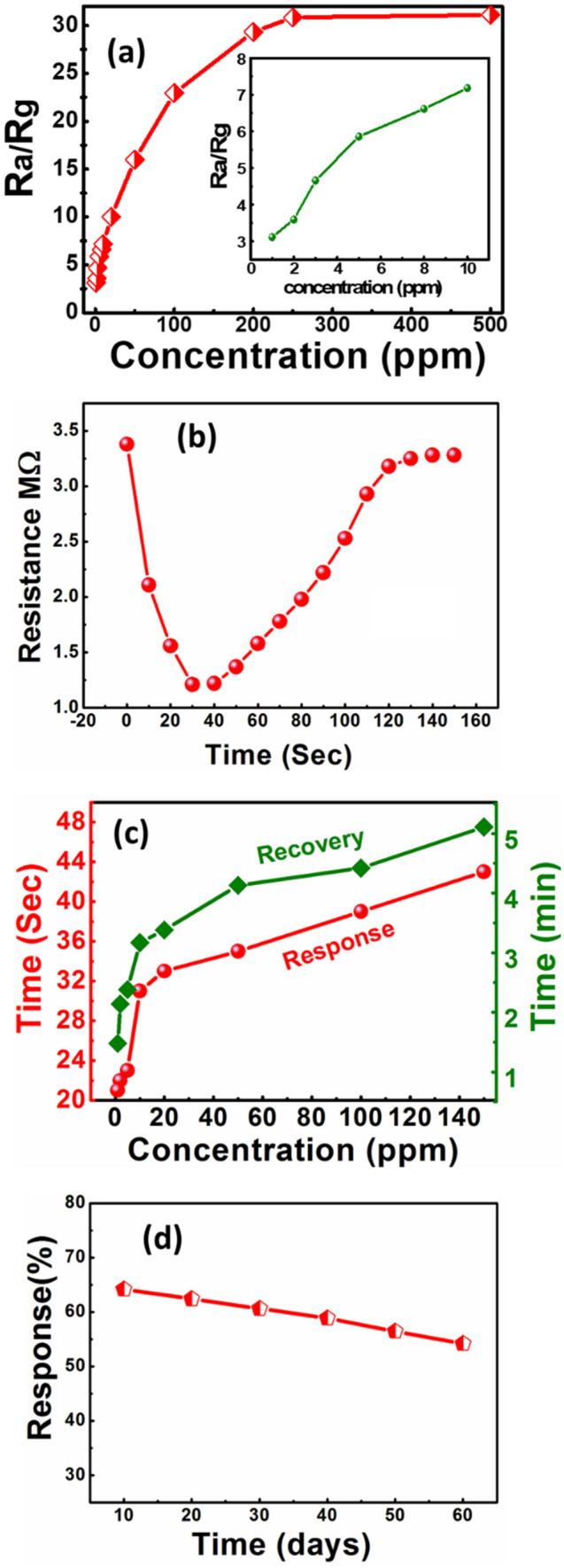
Response characteristics of sensor G2 with respect to -(**a**) the concentration of H_2_S at 300^○^C, -(**b**) Transient for 1 ppm H_2_S at 300^○^C, (**c**) Response/Recovery over H_2_S concentration, and (**d**) stability.

Figure 7a depicts the sensor G2 response with respect to H_2_S concentration at the optimum operating temperature (300 °C). At low concentrations of H_2_S (1 to 10 ppm), the sensor displayed a strong linear relation between the response and gas concentration. However, the active surface leads to saturation that causes a saturated response from 100 ppm of H_2_S concentration. The low population density of target gas implies to low surface reactions, and thereby the lower response. However, the surface reaction increases due to a large surface cover with increased gas concentration. The response saturates above certain concentration thresholds, and the active surface gets ceased for possible interaction of gas with the sensing material^19^. The sensor G0, G1, and G3 response as a function of function of H_2_S concentration at the optimum operating temperature (300 °C) is highlighted in the supplementary section (SI-V).

The transient reaction of sensor G2 in Fig. 7b shows the distinct valley in the transient response, which points out that the developed material is a good candidate for detecting H_2_S, as the peak response occurs relatively earlier (23 s) and it has faster recovery (75 s) time for 1 ppb of H_2_S. In Fig. 7c, the data indicates the specific gas concentration and the response/recovery time in such sensor. The sensors response gets quicker if an H_2_S concentrations get higher which is according to natural pattern.

The stability and reproducibility studies of the G_2_ sensor are depicted in Fig. 7d. Testing of the sensor stability and reproducibility were carried out at optimized operating temperature (300 °C) with measuring the sensor response to 1 ppm H_2_S concentration. After every ten days of initial measurements, gas sensing measurements are repeated over a duration of two months. It is observed that the sensor response is almost 85% of its initial measurement.

### Improved H_2_S sensing mechanism of RGO/WO_3_

**Scheme 2:**
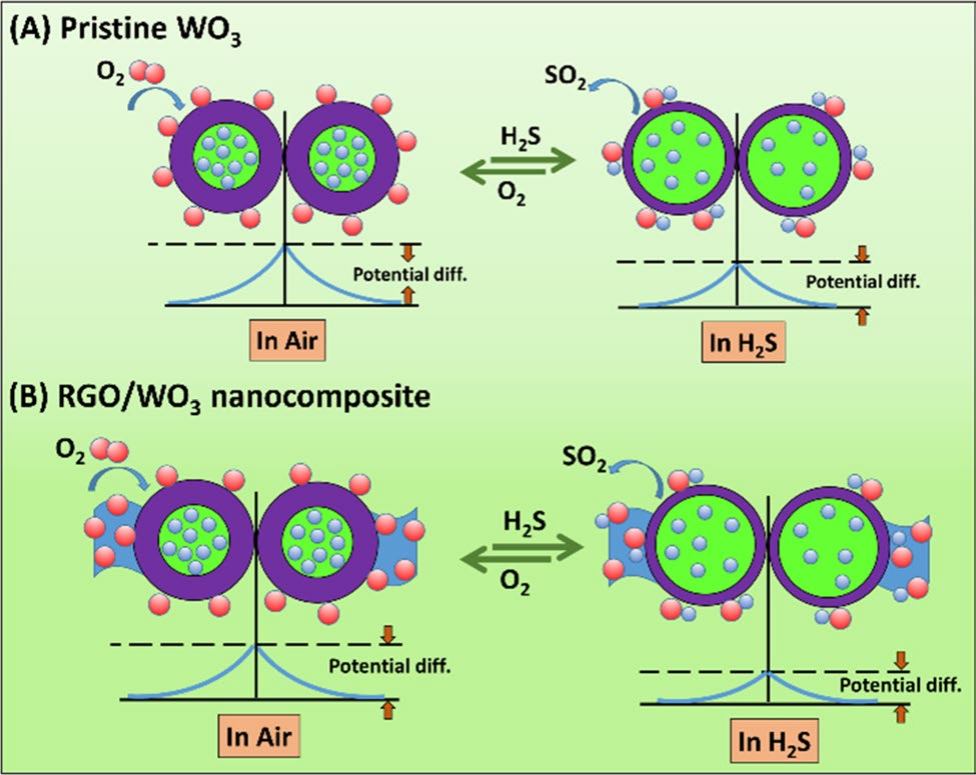
Schematic of (**A**) Pristine WO_3_ and (**B**) RGO/WO_3_ nanocomposite sensor system.

The surface adsorption and reaction models are described in Scheme 2, to illustrate the H_2_S sensing process. Tungsten trioxide is well known for resistive-type gas sensing material. The gas sensing response of WO_3_, is prominently influenced by the chemisorbed oxygen, type of the test gas, and the interaction between them. When the sensor temperature is around 300 °C, the dominant chemisorbed oxygen ion is O^−^^20^. As shown in the Scheme 2A, in the absence of H_2_S gas, the molecules of oxygen adsorbed onto the surface of WO_3_ nanoplates converted into O^2−^, O^−^ or O_2_^−^ by catching the electrons from the CB of WO_3_. Eventually, the electron depletion region is formed, which has comparatively larger resistance state^21^. When H_2_S gas is flowed over the sensor, the chemisorbed oxygen reacts with H_2_S molecules, thereby releasing the electrons back to CB of WO_3_ (see Eq. 2). This forms the lower resistance state of the sensor.

2 $${\text{H}}_{{2}} {\text{S}}_{{({\text{g}})}} + {\text{3O}}^{ - }_{{({\text{ads}})}} \to {\text{SO}}_{{{2}({\text{g}})}} + {\text{H}}_{{2}} {\text{O}}_{{({\text{g}})}} + {\text{3e}}^{ - }$$

As the working temperature rises up, even better response can be realized, due to more number of active oxygen molecules. This process can be preserved to a certain degree, then-after the gas adsorption becomes subsequently difficult and gas molecules starts to desorb greatly. This eventually, reduce the response performs of the sensor^22^.

There are many reasons for crediting the improved H_2_S sensing response by a 0.3 wt% RGO/WO_3_ nano-composite sensor (sample G2). First of all, the hierarchical mesoporous nanostructure enabled the better flow of H_2_S gas into the sensor through variety of channels, resulting in the reaction phase in a fine touch between H_2_S and internal WO_3_ grains. Secondly, the transfer of charge carriers get facilitated by RGO with excellent electrical properties. And thirdly, additional oxygen molecules on the surfaces of sensors from the WO_3_ conduction bands, and chemisorbed oxygen concentrations rises the primarily O^−^ as seen in Scheme 2b. More electrons get emitted into the sensing medium due to increasing the O^−^ concentration. This results in a higher H_2_S response in the composite system, than a pure WO_3_. WO_3_ is, moreover, a common semiconductor of n-type; and RGO is a semiconductor of p-type. The movement of majority charge carriers (holes and electrons) combines together, and results in the decrease in the effective carriers’ concentration, followed by vacant space charge region, called as depletion layer. This depletion layer is formed near the interface, which leads to increase the Ra value (resistance in air). Therefore, the response gets increased^23^. In the following, the comparison Table 3 is made to describe the present work status with the state-of-art literature.

**Table 3:** Comparison present work status with the state-of-art literature.

Material	H_2_S conc (ppm)	Working temp (°C)	Response (Ra/Rg)	Refs.
CuO-functionalized WO_3_ nanowires	100	300	6.72	^24^
WO_3_ nanowires	100	300	1.84	^24^
Pd-NPs/Pd-embedded WO_3_ nanofibers	1	350	1.36	^25^
0.01 wt% Au	2	300	7.5	^26^
Hexagonal (h-) WO_3_	10	200	~ 7 × 10^–9^	^27^
Ag-βVO_3_ nanowires	50	250	1.04	^28^
RGO/WO_3_	1	300	3.11	This work

### Photocatalytic activity study

Inexhaustibly available solar energy offers promising solution to solve the environmental problems^30–36^. Photocatalysis is one of the approaches to deal with the contaminants that are present in the water^37–42^. This novel approach motivated us to practice the as-developed RGO/WO_3_ nanocomposites for degradation of dye molecules in the water, under the natural sunlight in ambient conditions. For this purpose, Rhodamine B aqueous solution is prepared, to treat with the developed catalyst RGO/WO_3_. The resultant suspension is kept in the sunlight.

**Figure 8:**
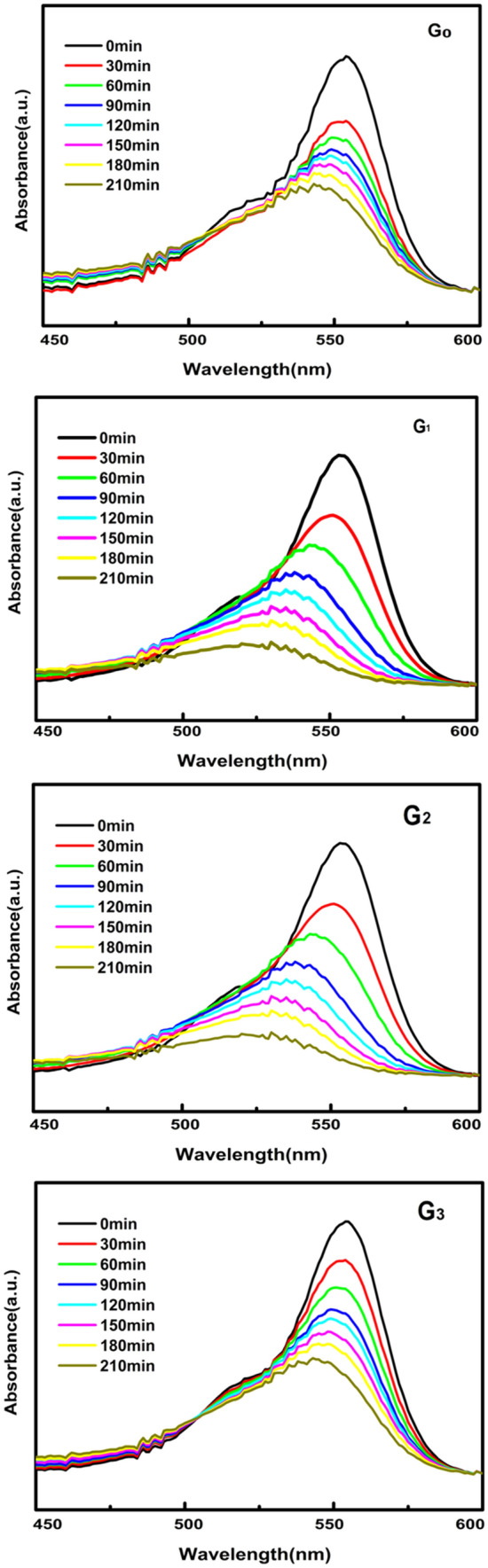
UV/Vis spectroscopic absorption of RhB aliquots in the presence of G0, G1 G2 and G3 catalysts.

Figure 8 describes the dynamic variations of Rhodamine-B absorption, in the presence of catalysts G0, G1, G2 and G3. It shows that with the increased irradiation time, the characteristic peak of absorption for RGO/WO_3_ aliquots was gradually reduced. The peak reduction is not significant in case of pristine WO_3_. This clearly shows that RGO has a major impact in improving the surface area (seen in the BET analysis), thereby, the photocatalytic dye degradation process. After 3 h of irradiation, for catalyst G1 the blue shift of the absorption peak is very small (not more than 14 nm after 180 min, 539 nm). However, the optimized RGO incorporation in WO_3_ has a major role in enhancing the photocatalytic process. A larger hypsochromic peak shift of 24 nm after 180 min (510 nm) due to de-ethylation of RhB, is observed in sample G2. Catalyst G_3_ exhibited a significant peak shift after 210 min of irradiation (similar to pristine WO_3_). All in all, the solution's maximum absorbing range has gradually changed between 554 and 499 nm. RhB N-deethylation is the result of the gradual hypsochrome shifts of absorption during radioactivity^29^. This hypochromatic shift in λ_max_ of RhB equals a gradual deethylation of RhB, giving N,N,N0-triethyl rhodamine (TER, 539 nm), N,N0-diethyllrhodamine (DER, 522 nm), N-ethyl-rhodamine (MER, 510 nm) and 498 nm rhodamine. The RhB molecule step by step loosens the ethyl groups to become stable products. Meanwhile, the dye solution is translated colourless from pink, suggesting that the porous RGO/WO_3_ products are good having photocatalytic activity.

**Figure 9:**
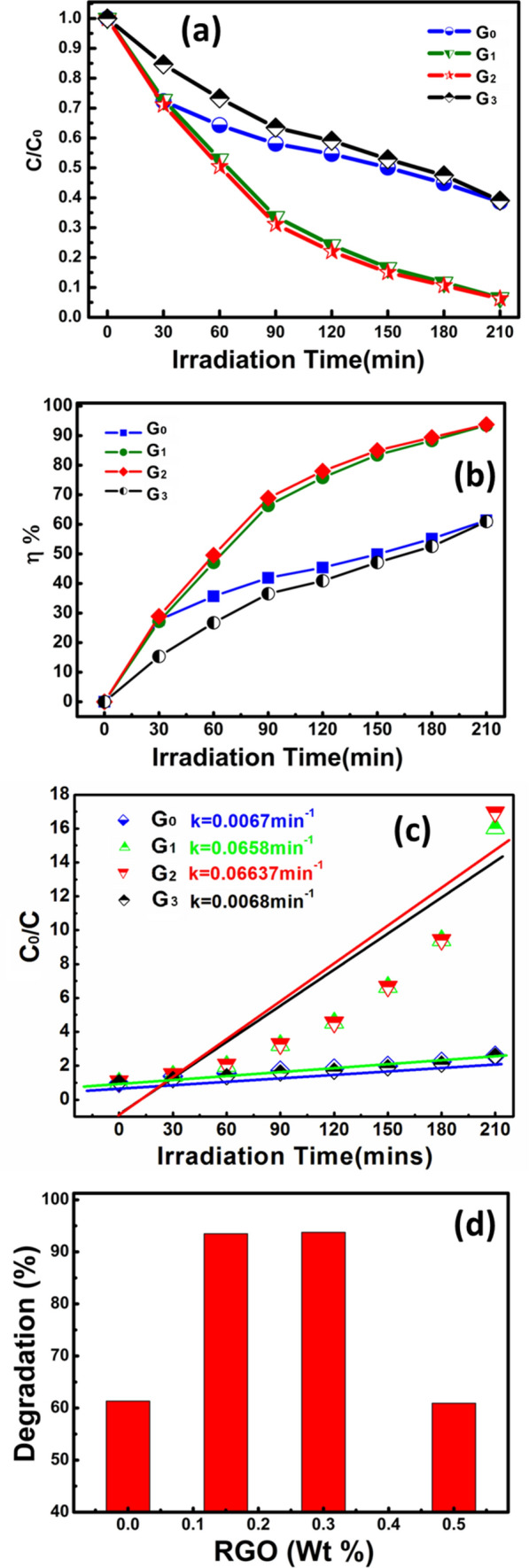
(**a**) Photo-activeness (C/C_0_), (**b**) Photo-degradation potency (η%) of pristine and RGO/WO_3_ catalysts under the natural sunlight for Rhodamine B dye molecules, (**c**) Photocatalytic degradation kinetics of developed catalysts, and (**d**) RGO incorporation effect over degradation after 210 min.

**Scheme 3:**
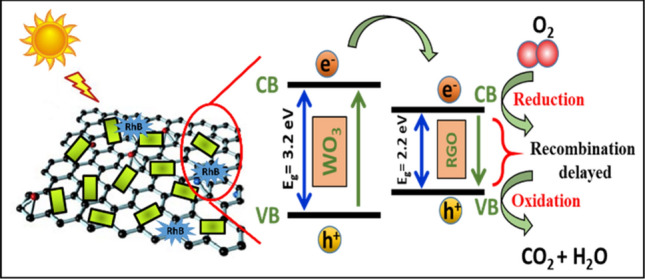
Schematic illustrating the photocatalytic activity of RGO/WO_3_ nanocomposite, under natural sunlight.

Figure 9a and b show the photo-activeness (C/C_0_) and photo-degradation potency (η%) of pristine and RGO/WO_3_ catalysts under the natural sunlight for Rhodamine B dye molecules. The sample G2 (0.3 wt% RGO/WO_3_) shows the highest degradation efficacy (94%) for RhB over the pristine WO_3_ (61%). This is due to the fact that pristine WO_3_ has low propensity to utilize the natural sunlight for photocatalysis. In addition, it suffers through high degree of charge-recombination. The role played by RGO in enhancing WO_3_ photoactivity can be attributed for the purpose of enhancing the separation and transfer of charge carriers from semiconductors (Scheme 3). It increases WO_3_′s light harvest capacity and promotes efficient transfer and separation of electron–hole photogenerated pairs. However, if the RGO weight percentage ratio is increased to 0.5%, photoactivity declines which can be attributable to the effect of shielding^43^. In the state of the art, such an optimum synergic result was widely observed between graphene and semiconductor^44–46^.

The curves for the entire sample sets are best fitted as shown in Fig. 9c, given the fact that dye degradation is indorsed to first order pseudo reaction with a simplified Langmuir–Hinshelwood model. The graphical evaluations of rate constants are increased from 0.0067 to 0.0664 min^−1^, which is nearly ten times more than pristine one. Figure 9d demonstrates the degradation efficiency pathway for RGO incorporation in weight percent. Pristine and 0.5 wt% RGO/WO_3_ exhibited lower degradation efficiency compared to 0.15% and 0.3% of RGO incorporation. The 0.3% of RGO have maximum hypsocromic shift followed by formation of Rhodamine from Rhodamine B with maximum degradation efficiency. The result can be correlated with the surface area of the photocatalyst. Herein, G2 with the higher surface area has provided more active sites for the reactant molecules. Large BET surface area with optimal RGO, holds strong adsorption ability to improve the photocatalytic activity. In the following, the comparison Table 4 is made to describe the present work status with the state-of-art literature.

**Table 4:** Comparison of present work status with the state-of-art literature.

Sample	Dye used	Degradation efficiency (%)	Reaction time	Refs.
c-WO_3−X_ /WO_3_ H_2_O	MB	~ 70	180	^47^
Ag WO_3_	AR 88	~ 29	180	^48^
Ag/CuO/WO_3_	AR 88	~ 62		^48^
Pure WO_3_	RhB	60	180	^49^
Mo WO_3_	RhB	92	180	^49^
Pristine WO_3_	RhB	28	210	^1^
Ru WO_3_	RhB	84	210	^1^
WO_3_	RhB	61	210	This work
RGO/WO_3_	RhB	94	210	This work

## Conclusion

In conclusion, the pristine and RGO incorporated WO_3_ hierarchical marigold micro-flowers are developed via simple hydrothermal route. The self-made RGO using modified Hummer’s method has dramatically improved the surface area of the parent metal oxide, by the factor of 7 (from 3.2 to 20.0 m^2^/g). XRD analysis confirmed the orthorhombic crystal structure of as developed RGO/WO_3_ nanocomposites. The morphological and EDS elemental mapping showcased the uniform spreading of WO_3_ on the graphene layer. The prime motivation of increasing the surface area and surface active sites was realized through BET analysis, which gave the hint to use the developed nanocomposites of multidisciplinary applications. The excellent ability of gas sensor towards H_2_S gas (Ra/Rg = 3.1 per ppm) and natural sunlight-driven photocatalysis of rhodamine B (94% in 210 min) were induced in the pristine WO_3_ by incorporating 0.3 wt% of RGO. Sample G1 and G2 almost have the almost same photocatalytic activity but different sensitivity. The gas response amongst the same metal oxide but with different loading of RGO, differs due to spillover mechanism. The work showcase, the designed approach of developing RGO/WO_3_ nanocomposite has high potential in multidisciplinary applications such as gas sensor and photocatalysis.

## Supplementary Information

Supplementary Information.
