# Supplementary material for: RGO/WO3 hierarchical architectures for improved H2S sensing and highly efficient solar-driving photo-degradation of RhB dye
Source: Sci Rep. 2021 Mar 3;11:5023. doi: 10.1038/s41598-021-84416-1 (PMC7930058; doi:10.1038/s41598-021-84416-1)
Supplement: Supplementary file 1 — Supplementary Information. [file 41598_2021_84416_MOESM1_ESM.docx]

**RGO/WO_3_ Hierarchical Architectures for Improved H_2_S Sensing and Highly Efficient Solar-Driving Photo-Degradation of RhB Dye**

Swati S. Mehta,^a,1^ Digambar Y. Nadargi,^a,1*^ Mohaseen S. Tamboli,^b^ Thamraa Alshahrani,^c^ Vasudeva Reddy Minnam Reddy,^b^ Eui Seon Kim,^b^ Imtiaz S. Mulla,^d^ Chinho Park^b^* and Sharad S. Suryavanshi^a^*

^a^ School of Physical Sciences, PAH Solapur University, Solapur (M.S.) 413255, India.

^b^ School of Chemical Engineering, Yeungnam University, 280 Daehak-ro, Gyeongsan, 38541, Republic of Korea

^c^ Department of Physics, College of Science, Princess Nourah Bint Abdulrahman University, Riyadh 11671, Saudi Arabia

^d^ Former Emeritus Scientist (CSIR), Centre for Materials for Electronics Technology, Pune-411008, India.

^1^ These authors contributed equally.

email: Dr. Digambar Nadargi ([digambar_nadargi@yahoo.co.in](mailto:digambar_nadargi@yahoo.co.in)), Prof. Chinho Park ([chpark@ynu.ac.kr](mailto:chpark@ynu.ac.kr)), Prof. Sharad Suryavanshi ([sssuryavanshi@rediffmail.com](mailto:sssuryavanshi@rediffmail.com))

**Contents**

**1. Supporting information SI-I:** XRD profiles of samples G_0_, G_1_, G_2_, and G_3_.

**2. Supporting information SI-II:** Table S1: EDAX elemental analysis of all the samples (G_0_, G_1_, G_2_, and G_3_)

**3. Supporting information SI-III:** EDS spectrum of sample G_0_, G_1_, and G_3_.

**4. Supporting information SI-IV:** FESEM images showing the loosening of microflower compactness.

**5. Supporting information SI-V:** Response as a function of H_2_S concertation for sensors G_0_, G_1_, G_2_, and G_3_.

**Supporting information SI-I**

**
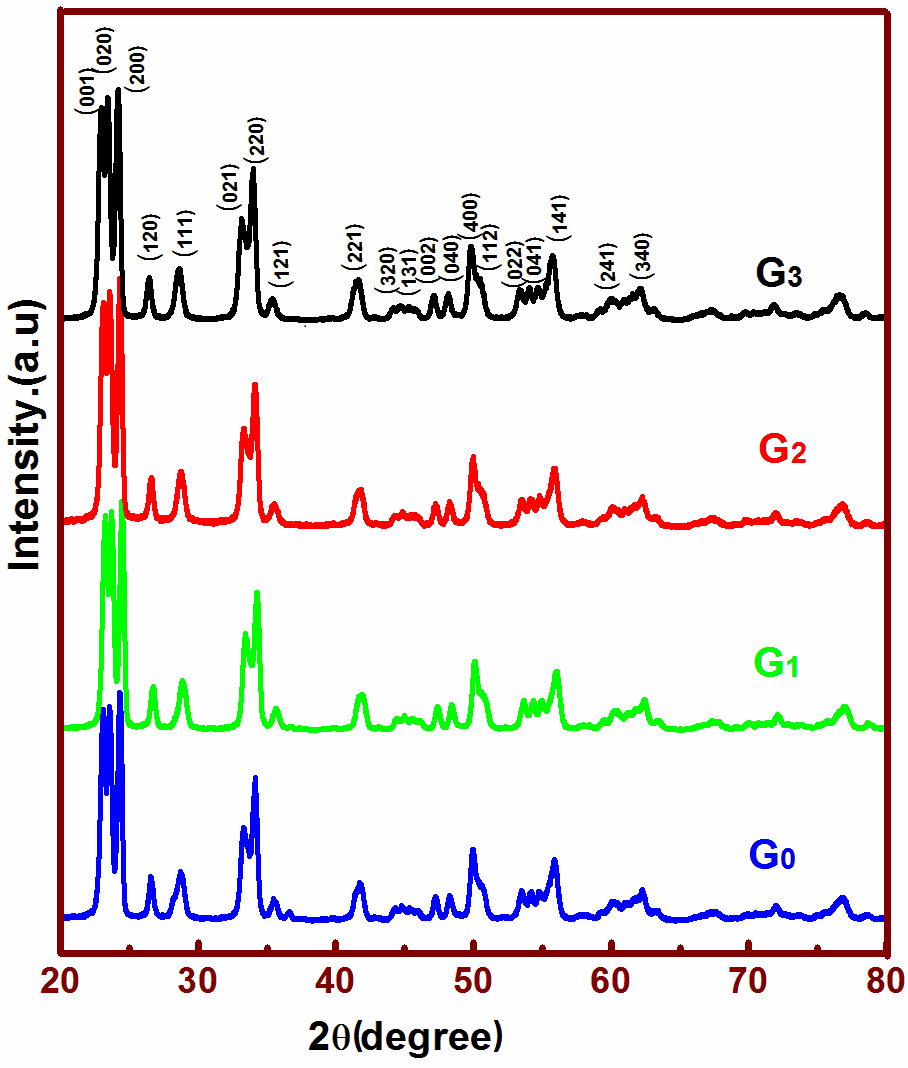
**

Figure S1: XRD profiles of samples G_0_, G_1_, G_2_, and G_3_.

**Supporting information SI-II**

Table S1: EDAX elemental analysis of all the samples (G_0_, G_1_, G_2_, and G_3_)

| **Sample code** | **Element** | **Weight%** | **Atomic%** |
| --- | --- | --- | --- |
| GO  (Graphene oxide) | C  O | 63.27  31.72 | 71.13  26.76 |
| G_0_ | W  O | 80.64  16.85 | 25.79  61.92 |
| G_1_ | W  O  C | 79.52  16.33  4.15 | 24.04  56.75  19.21 |
| G_2_ | W  O  C | 79.21  15.28  5.51 | 23.35  51.78  24.87 |
| G_3_ | W  O  C | 76.46  17.03  6.51 | 20.56  52.62  26.82 |

**Supporting information SI-III**


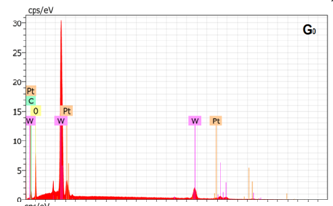


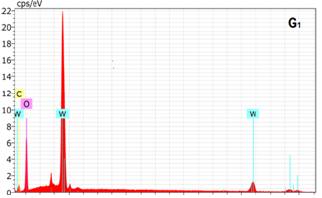


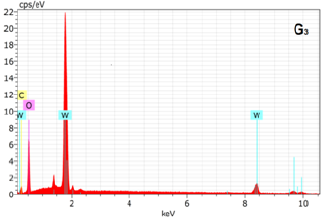


Figure S2: EDS spectrum of sample G_0_, G_1_, and G_3_.

**Supporting information SI-IV**


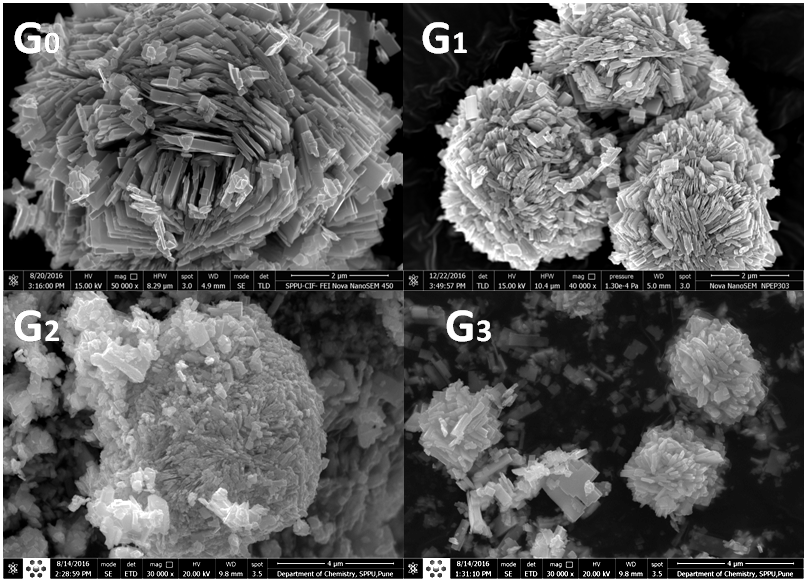


Figure S3: FESEM images showing the loosening of microflower compactness.

**Supporting information SI-V**


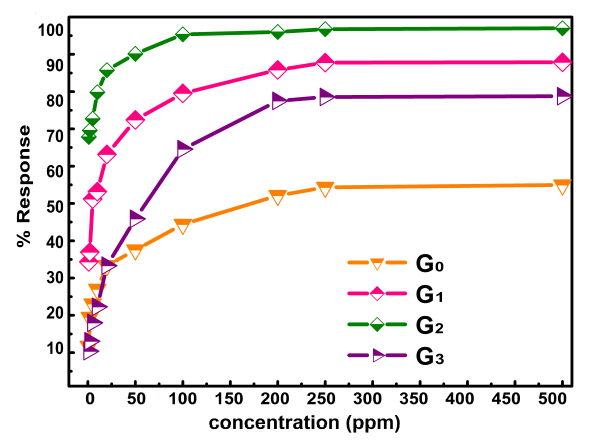


Figure S4: Response as a function of H_2_S concertation for sensors G_0_, G_1_, G_2_, and G_3_.
